# Cooperation and Social Rules Emerging From the Principle of Surprise Minimization

**DOI:** 10.3389/fpsyg.2020.606174

**Published:** 2021-01-21

**Authors:** Mattis Hartwig, Achim Peters

**Affiliations:** ^1^Institute of Information Systems, University of Lübeck, Lübeck, Germany; ^2^Clinical Research Group, Brain Metabolism, Neuroenergetics, Obesity and Diabetes, University of Lübeck, Lübeck, Germany

**Keywords:** free energy, social rules, decision making, cooperation, surprise minimization

## Abstract

The surprise minimization principle has been applied to explain various cognitive processes in humans. Originally describing perceptual and active inference, the framework has been applied to different types of decision making including long-term policies, utility maximization and exploration. This analysis extends the application of surprise minimization (also known as free energy principle) to a multi-agent setup and shows how it can explain the emergence of social rules and cooperation. We further show that in social decision-making and political policy design, surprise minimization is superior in many aspects to the classical approach of maximizing utility. Surprise minimization shows directly what value freedom of choice can have for social agents and why, depending on the context, they enter into cooperation, agree on social rules, or do nothing of the kind.

## Introduction

The “free energy principle” rests upon the assumption that all cognitive systems strive to minimize surprise, i.e., minimize their uncertainty about future outcomes (Friston, 2005, 2010). Being unable to avoid surprises can cause stress which, if it persists, may become toxic, leading to coronary heart disease, depression and type 2 diabetes (Peters et al., 2017). Originally being introduced as a principle for dealing with perception and action, the free energy principle has been applied to more complex domains like decision making and exploration (Schwartenbeck et al., 2015).

In this article, we apply the concept of surprise minimization more broadly to a multi-agent situation where agents can communicate and consider the option of cooperation. Intuitively everyone knows that cooperation with other people can be a good way to avoid stress. We show that according to the principle of minimizing surprises, in some cases the agents actually choose to cooperate, but in other cases they do not.

The article is structured as follow. In Section “Surprise minimization as an extended model of classical maximization of expected utility” we introduce the underlying concepts and metrics. Section “The role of surprise minimization on the emergence of social rules” covers the multi-agent case and shows under which circumstances cooperation occurs and under which not. Section “Discussing potentials of surprise minimization for sociology and medicine” discusses the impacts of our conceptual analysis and argues why it is superior to the pure form of utility maximization, which is often used to explain human behavior and decision making. Section “Conclusion” summarizes the final conclusions.

## Surprise Minimization as an Extended Model of Classical Maximization of Expected Utility

### Bayesian Brain – Surprise Minimization

The expectation concept plays a central role in Bayesian statistics. The aspect that the fulfilment or disappointment of expectations flows recursively back into the expectation itself has already been modeled mathematically and integrated into modern brain research. In the 18th century, Thomas Bayes defined probabilities as reasonable expectations (Bayes and Price, 1763; Cox, 1946). Environmental states *S* have a certain probability of taking a specific value denoted as *P*(*S* = *s*). Values for probabilities range from 0 (impossible) to 1 (reliable). Bayesian probability theory introduces the concept of a prior probability (or prior expectation) denoted as *P*(*S*) over a state vector *S*, which combines previous knowledge and basic assumptions of the observer in a probability distribution of *S*. The key idea of Bayes’ theorem is that the prior probability distribution is updated by means of new evidence (data, observations), thereby creating a posterior probability distribution denoted as *P*(*S*|*O* = *o*), where *O* denote the observed states and *o* denote the observed values for the states. The posterior distribution can be calculated using the Bayes’ theorem:

$$P\left(S|O=o\right)=\frac{P\left(O=o|S\right)P\left(S\right)}{P\left(O=o\right)}$$

The term *P*(*O* = *o*|*S*) is the likelihood of the observation given the state S, and the *P*(*O* = *o*) is the prior for making the observation *O=o*. As the Bayes’ theorem is central to modern theoretical brain research, the neurobiological concept is called the Bayesian Brain model.

It was the psychiatrist and physicist Karl Friston from University College London who developed the model further over the last 20 years (Friston, 2012). The “free energy principle” rests upon the fact that self-organizing biological agents resist a tendency to disorder and must therefore minimize the entropy of their sensory states (Friston, 2005, 2010). Free energy is a term from information theory, where it is referred to as variational free energy. The variational free energy and the free energy of stochastic thermodynamics not only share the same mathematical formalism, but are also closely related to each other on a deeper level (Sengupta et al., 2013; Kiefer, 2020; Parr et al., 2020), but this will not be discussed any further here. The information-theoretic variational free energy bounds surprise, and is conceived as the difference between an organism’s predictions about its sensory inputs (embodied in its internal model of the world) and the sensations it actually encounters. Reducing “free energy” inevitably reduces “surprise” – as measured by a violation of predictions. Likewise, reducing “expected free energy” inevitably reduces “expected surprise” – known as entropy or uncertainty. Thus, in the long-term, agents are all compelled to avoid surprises and resolve uncertainty.

Accordingly, prior beliefs are iteratively updated in the Bayesian brain on the basis of new evidence. Future surprises or prediction errors can be minimized through two processes: perceptual inference that updates the prior expectation into a posterior probability distribution, and active inference that alters sensory input through actions that change the agent’s position in space.

#### Perceptual Inference

The perceptual aspect of the Bayesian Brain is often referred to as predictive coding. A basic formulation of the problem of perceptual inference is in terms of cause and effect. States in the world have effects on the brain, processes in the world are the causes for sensory input. The problem with perception is to use the effects – the sensory data to which the brain has access – to find the causes (Hohwy, 2013). It is not easy to infer from only the known effects back to their hidden causes, because the same effect can arise from many different causes.

Technically speaking, there are two methods of performing Bayesian inference. The first is exact Bayesian inference, using the explicit formula of the Bayes theorem, in which a prior belief is directly updated into a posterior belief. The second is the approximate Bayesian inference, which has been studied for decades in the field of machine learning and later been transferred to predictive coding. The approximate method uses as one important component a “generative model,” in order to be able to infer from an effect its probable cause. The generative model consists of a “prior” belief and a “likelihood” allowing predictions about what sensory information will arrive. Thus, the generative model can predict an effect, given the cause that is considered most likely. Simply put, the model can create an effect from a cause. If the prediction does not match the sensory input, the agent will be surprised. Surprise (which, as said, can be approximated by free energy) can be estimated with the help of the so-called prediction error, which is the difference between the predicted and the actual sensory input. The prediction error is used in turn to update the prior belief by transforming it into an approximate posterior belief. Although the mathematical procedure for exact Bayesian updating and the procedure used for predictive coding are formally different, they lead essentially to the same results.

Bayesian inference describes the minimization of variational free energy, where free energy is the overall amount of prediction error. There is evidence that both perceptual inference and learning can be described as a minimization of prediction errors (i.e., free energy; Rao and Ballard, 1999; Friston, 2005). The free energy concept, originating from statistical mechanics, makes it possible to transform difficult integration problems (which occur when Bayes’ rule is applied directly) into a more treatable optimization problem (e.g., prediction error minimization). Free energy can be regarded as the information a person is lacking, and which he/she could use to make his/her internal model as close as possible to reality. Thus, Bayesian inference, as used in perception, reduces our uncertainty about the states of affairs in the world that have caused our sensations.

The structural and functional brain networks are organized hierarchically (Park and Friston, 2013). The lower cortical areas are nearer the primary sensory input, the higher areas play an associative role. Due to its hierarchical architecture, the brain can learn both its own priors and the intrinsic causal structure of the world that generates the sensory input. In hierarchical Bayesian inference, the mid-level priors now become “empirical priors.” This follows because they become accountable to empirical (sensory) data and can therefore be optimized to minimize prediction errors at each hierarchal level. Neuroanatomical hierarchy means distinguishing between bottom-up and top-down connections (Salin and Bullier, 1995). In the auditory and visual system, it has been shown that of bottom-up prediction errors and top-down predictions, the latter are particularly important (Chennu et al., 2013; Dijkstra et al., 2017). Using dynamic causal modeling of mismatch responses, elicited in an oddball paradigm, Garrido and coworkers showed that the late components of event-related responses were mediated by top-down connections (Garrido et al., 2007). This work demonstrated that top-down connections are necessary for recurrent interactions among levels of cortical hierarchies.

#### Active Inference

Active Inference is the second way how to minimize prediction errors. In perceptual inference, agents strive to update their internal model of the world, whereas in active inference, agents change their environment in order to better inform their beliefs about the world (Adams et al., 2013). In active inference, sensations can be changed by action in such a way that they become more like the predictions.

In the visual and auditory system, evidence supports that the systems’ functioning can be attributed to active inference (Chennu et al., 2013; Kok and de Lange, 2014). In the motor system, actions have been shown to effectively minimize proprioceptive prediction errors; in the simplest case, motor reflexes can accomplish such a minimization (Adams et al., 2013). In the visceromotor system, the internal body environment is subject to allostatic (predictive) regulation (Sterling, 2012), which reduces viscerosensory prediction errors (Barrett and Simmons, 2015). In this way, perceptual and active inference can minimize the surprise of living beings like ourselves.

### Surprise Arises When an Agent Reaches a State That Deviates From His Goal States

A well-defined agent to exist must occupy a limited repertoire of states. For a fish it could be a binary state *S* environment that could be either in the water (unsurprising) or out of the water (surprising; Friston, 2009). Information theory describes the violation of predictions as surprise, self-information, or surprisal (Tribus, 1961). The less likely it is that an agent occupies a particular condition, the greater the surprise is when the condition will actually be reached. States can be either discrete, if only a fixed number of values are possible to the state (e.g., in or out of the water) or continuous, if there are unlimited possible values (e.g., water temperature). In the latter we have a probability density function over the infinite possibilities.

Since the brain is hierarchically organized (Felleman and van Essen, 1991; Swanson, 2000), there are priors coded at different levels. Prior expectations encoded at higher levels change (if at all) only over a long period of time and have a high level of abstraction (e.g., rules, principles). When we talk about goal states we mean states, that have a high value in the high-level prior distributions (i.e., are encoded with high precision). In contrast, prior expectations encoded at lower levels are updated much more frequently and have a low level of abstraction (various details; Friston, 2005). The lower-level prior expectations are closer to the level of the incoming sensory input (in the retina of the eye, in the thalamus) and are therefore constantly adapted to rapid changes in the environment. Among the high-level priors are the goal expectations of a person, for example social and moral attitudes (fairness, justice, and honesty) and individual goals (at work, family, and partnership; Peters et al., 2017).

The beliefs about the “states that agents believe they should occupy” involve regions such as the ventromedial prefrontal cortex prefrontal and the orbitofrontal cortex (Bechara et al., 2000; Gottfried et al., 2003; O’Doherty et al., 2003; Roesch and Olson, 2004; Barron et al., 2015). Such regions play a key role in setting the expected value (i.e., free energy) of future states. These goal or prior preferences provide a neurally coded reference point for goal-oriented behavior. With neural coding we refer to the idea that beliefs (e.g., preferences) are represented as probability distributions over states of the world specified by their sufficient statistics, which we assume are encoded in the synaptic network of a hierarchically organized brain structure. Which probabilistic neural code is used for this is not clear yet, but the Laplace approximation is a promising candidate, because it can efficiently treat continuous and correlated states; it is particularly efficient because it specifies recognition densities completely by their mean; the only other sufficient statistic (the conditional precision) can be derived from the mean and does not need to be explicitly coded (Friston, 2009). In many cases, capturing the goal states requires several dimensions, so that the probability distribution over these states becomes multi-dimensional. For instance, people who want to buy a car usually have multidimensional goals (e.g., size, price, brand, and ecological sustainability, …).

In summary, an agent is surprised when it reaches a state that deviates from his goal state. But if he succeeds in minimizing long-term surprise, he automatically assumes conditions that are optimal for maintaining his physical, mental and social well-being.

### The Risk of a Policy Indicates the Likelihood of Being Surprised in the Future

Decision-making can also be treated as an inference problem (Friston et al., 2013). The agent infers which of his strategies or policies is most promising for achieving his goal states. Policies can be either individual actions or sequences of actions depending on the context, here denoted as π. In this inference process, the agent uses a generative model to make predictions about which states can be reached by using a particular policy denoted as *P*(*S*|π). In other words, the generative model predicts future scenarios for each policy.

**FIGURE 1:**
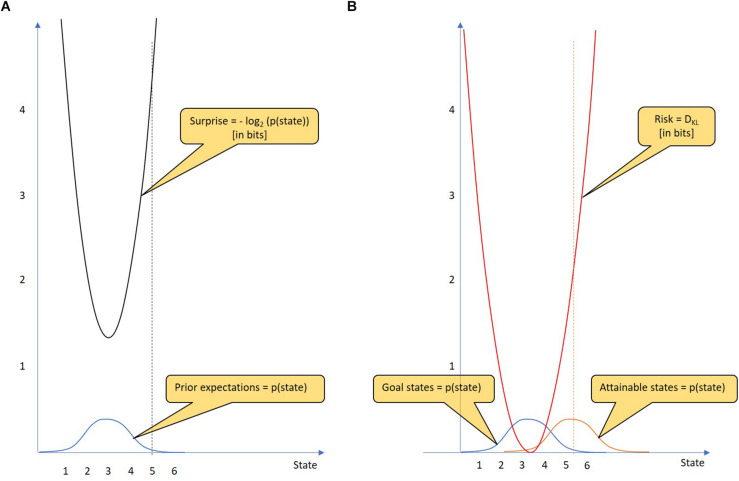
Goal states, surprise and risk. **(A)** Surprise: The goal states of an agent are represented by a prior probability distribution (blue) that indicates how much the agent prefers particular states of the world. From the state reached by the agent, the resulting surprise (black) can be calculated using the logarithm of the state’s probability (which is given by the goal state probability distribution). The unit of surprise is “bit.” The surprise is minimal when the agent reaches the most preferred goal state (state 3), whereas the surprise increases the more the state reached deviates from the most preferred goal state. For example, a 4.21-bit surprise occurs when the agent reaches state 5. Thus, an agent that minimizes surprise most likely takes on the conditions that he believes should be occupied. **(B)** Risk: In decision-making, a policy should be selected from a given policy repertoire. A key step in decision-making is assessing the risk of each policy. For each policy, predictions are made as to which states can be reached. These attainable states are represented by a probability distribution over attainable states (orange). The risk of a policy can be assessed by the Kullback-Leibler divergence (see text). As the mean of the attainable states changes, so does the risk (red). For example, if the mean of the attainable states is state 5.3, the risk of this policy is 2 bits.

Figure 1A shows an example of a Gaussian prior distribution over a continuous state variable. When the agent enters a particular state (state 5), the degree of surprise is clearly determined (4.21 bits). The problem is more difficult with future predictions. It is not certain that a particular policy will result in only one defined state. Instead, using a particular policy, there is a probability distribution over attainable states. Figure 1B shows two probability distributions, that over goal states and that over attainable states given the chosen policy. The risk of a policy is defined as the Kullback-Leibler divergence *D*_*KL*_ between the two probability distributions. The Kullback-Leibler divergence is defined for continuous and discrete probability distributions. While the continuous uses an integral over the infinite state values, the discrete uses a sum. In the following we will work with the discrete version of the measure:

(1) $$D_{KL}=-\sum_{S}P\left(S|\pi \right)\log_{2}\frac{P\left(S\right)}{P\left(S|\pi \right)}$$

The Kullback-Leibler divergence is a mathematical tool that measures the “distance” between any two probability distributions (although it is not a metric measure). In simplified terms, *D*_*KL*_ is a measure of future surprise, with diverse applications such as applied statistics, machine learning and neuroscience. The larger the distance between goal states and attainable states is, the larger is the *D*_*KL*_ and thus the risk of the policy in question. Remarkably, the risk increases quadratically with distance (given Gaussian distributions with equal variance). In Figure 1B, the risk of the policy is 2.00 bits.

The beliefs about the “states that can be reached” involve regions like the pre-supplementary motor area (pre-SMA; Rushworth et al., 2004; Nguyen et al., 2014). From this perspective, the pre-SMA includes a generative model that predicts outcomes that can be achieved with alternative policies (policy 1, policy 2,…, policy n; Friston et al., 2013). All previous experiences throughout life have formed the current prior beliefs that underpin how predictions or decisions are made in the Bayesian brain. For example, child adversities or failed attachment to parents, but also every strong positive or negative experience in school, work and private life, influence the way policies are chosen in the Bayesian brain to ensure future well-being. Amygdala- and hippocampus-dependent emotional and declarative memories shape the generative model in a lengthy iterative process. No person in this respect is free from such biographical biases toward the prediction of future events.

Among the various brain regions, there is a candidate region capable of integrating beliefs about attainable states and goal states – the anterior cingulate cortex (ACC; Lee et al., 2007; Nguyen et al., 2014). The ACC is in an ideal position to assess and compare the risks of alternative policies and to enable decisions-making under uncertainty (Paulus et al., 2002; Feinstein et al., 2006; Behrens et al., 2007; Sarinopoulos et al., 2010; Karlsson et al., 2012; Liljeholm et al., 2013). Electroencephalography studies highlighted the presence of specific markers of performance monitoring in the time (error related negativity) and time frequency domain (theta synchronization) that originate from the ACC (Falkenstein et al., 1991; Cavanagh et al., 2009). Interestingly, the same markers of the activity of the performance monitoring system seem to be present when observing someone else performing an error, or when interacting with someone else, when monitoring of its movements is needed (de Bruijn et al., 2007; Moreau et al., 2020).

Activation within the ACC has been interpreted by some researchers as reflecting selection of action (Posner et al., 1988) and by others as conflict monitoring (Botvinick et al., 1999) – which is not mutually exclusive. A complementary perspective provides evidence for an “expected risk model” of the ACC, which indicates that the ACC assesses the expected risk of a given policy (Brown and Braver, 2007). The monitoring processes mentioned here cluster primarily in the transition zone between the cingulate and paracingulate (areas 24 and 32), association (area 8), and premotor cortices (area 6), an area that has extensive connections to brain areas involved in the control of cognitive and motor processes and the regulation of autonomous arousal (Paus, 2001; Critchley et al., 2003). These interactions allow the ACC to signal the need for performance adjustments when decisions are uncertain (Ridderinkhof et al., 2004).

In engineering and optimal control theory, the process of minimizing the divergence between predicted and preferred states is called KL control. Basing beliefs about future choices on KL divergence is formally related to optimization schemes based on KL control; particularly risk sensitive control. KL control is related to what neurobiologists call goal-directed processes. Goal-directed processes assess an expected *value* of one or more options for action in a given situation. In the typical approach describing goal-directed processes, the expected value is “expected utility” (Moors et al., 2017; Moors et al., 2019), but it can also – in the strict sense of KL control – be “expected free energy” (Friston et al., 2013). Depending on the approach used, goal-directed processes can maximize utility, or minimize free energy (i.e., surprise). Whatever the case, the action option with the highest expected *value* activates its corresponding action tendency, and this action tendency can be manifested in overt behavior.

In summary, an important aspect in resolving uncertainty in strategy selection is to reduce the expected surprise with regard to one’s own goals. In this respect, risk assessment and the comparison of risks between policies are important steps in the decision-making process. The resulting basic rule says that an agent with a given policy repertoire is well advised to choose the policy with the least risk.

### A Low-Risk Policy Has High Expected Utility and Leaves Many Options (Attainable States) Open

A good policy is able to balance environmental exploitation and exploration. Exploitation is the method of choice as long as the accessible source is rich. In this case, only expected utility is maximized. Exploration is useful when there is no immediate access to resources. However, if an agent exploits a rich source, he should look for other options that may become important when the current source is drying up. This means that – in some contexts – agents are compelled to seek novel states whereas in other contexts they maximize expected utility.

Eq. 1 can be decomposed into two components (Schwartenbeck et al., 2013):

(2) $$D_{KL}=-\sum_{S}P\left(S|\pi \right)\log_{2}\text{P}\left(\text{S}\right)+H\left[P\left(S|\pi \right)\right]$$

The first term represents the expected utility over outcomes that depends on an agent’s priors and constitutes the goals of the agent, i.e., his beliefs about the utility of final states. A reduction of the first term of the *D*_*KL*_ is accompanied by a reduction of the policy’s risk and thus ensures the outcome with the highest expected utility. The second term is the entropy *H* over attainable states that reflects the number of different outcomes that the agent is likely to experience under the particular policy. Increasing the entropy over attainable states means that the agent expects to keep many options (attainable states) open. Under certain circumstances, the surprise can be minimized when an agent selects a policy that increases the likelihood of visiting new states. Thus, surprise minimization can be considered an extension of the classical maximization of expected utility.

### Restrictive Social Rules or Laws That Exclude Reaching Certain States May Increase or Decrease the Risk, Depending on the Context

Social rules or constraints can intervene at two points in the decision-making process: First, rules can alter the probability distribution over the attainable states for a policy, and in so doing they change the risk of that policy; second, rules can limit the repertoire of policies, thereby changing the certainty in policy selection. Here, we show that, depending on the context, social rules can have both good and bad effects. For example, adhering to a calorie restriction diet – which is a life-style constraint – limits the number of attainable states: e.g., eating ice cream is excluded. Sometimes such constraints are useful [e.g., smoking cessation (Anthonisen et al., 2005)], sometimes they are ineffective or even harmful [e.g., calorie restriction diet (Villareal et al., 2006; Look-Ahead-Research-Group, 2013)].

Basically, restrictions that make certain states unreachable diminish the value of a policy because such constraints reduce the number of open options (Eq. 2). There are two possible ways in which restrictions may affect the risk of a policy. First, if the restriction precludes a highly undesirable condition that is likely to occur, the gain in expected utility is large. Such a large utility gain would outweigh the disadvantage of limiting open options, making the policy attractive.

Second, the gain in expected utility is low if constraints prevent the achievement of a condition that is moderately desirable and moderately likely. In such a case, the foreseeable loss of open options (achievable conditions) outweighs the small gain in expected utility, making the policy unattractive.

In conclusion, there are cases where restrictive rules or constraints are on the whole favorable, although they limit open options. In contrast, there are other scenarios in which restrictive rules or constraints pose a high risk, mainly because they prevent the agent from exploring the world. The concept of surprise minimization, therefore, by no means precludes agents from active exploration or appreciating novelty but rather explicitly predicts that this is an important factor in guiding our behavior. In the next section we will look at how such social rules or constraints could emerge when two agents are in the game.

## The Role of Surprise Minimization on the Emergence of Social Rules

### For Two Agents, the Mutual Agreement to Limit the Number of Policies at Choice May Reduce Uncertainty

In this section, we examine decisions on whether agents should cooperate or not. In particular, it will be asked whether the principle of surprise minimization provides a basis for an agent’s decision to cooperate. Cooperation is defined here as the willingness to introduce and accept social rules, an interaction that we clearly distinguish from a unique friendly commitment. Cooperation in this sense, as well as friendly and hostile commitments, are all dealt with later in our example. Like the sociologist Niklas Luhmann we here consider interactions basically microsociological (Luhmann, 1987). Luhmann stated that double contingency in democratic reality is overcome by the developing individuality and ability to communicate. Through observation of the other as well as through trial and error, an emergent order arises in the course of time, which Luhmann calls social system.

In the interaction between the two agents “Ego” and “Alter,” the states *S* of the system depend on the policies that each of the agents chooses. In the communication between Alter and Ego, information is exchanged that is used in both agents to change neuronally coded expectations *P*(*S*|*m*) where *m* is referring to a specific agent. Luhmann emphasizes in this context that in the characterization of those systems that can acquire and process information, another feature must be added that indirectly serves to define the concept of information: these must be self-referentially operating systems, i.e., systems that must always participate in the change of their own states (Luhmann, 1987).

**FIGURE 2:**
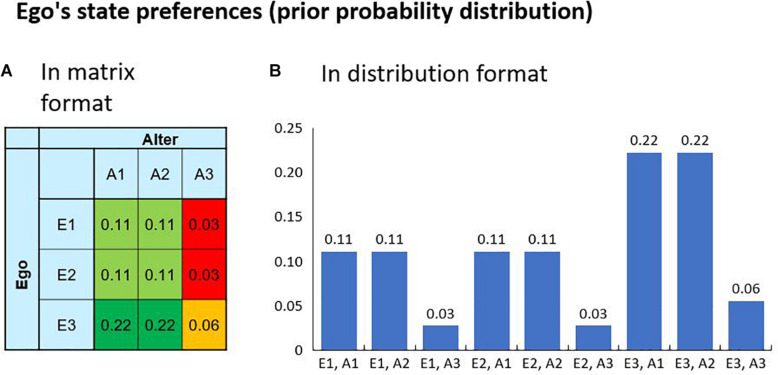
The agent’s preferences over the possible outcomes are encoded in a prior probability distribution (here 9 possible states). The preferences can be visualized in a decision matrix **(A)** or in a bar chart **(B)**. A high value is associated with a high preference and a low value with a low preference. Given the nature of a probability distribution, the maximum preference is one. If one state has the probability of one, the agent will only accept this state as a goal and the realization of all other states would result in maximum surprise. In our example, the Ego agent has high preference for performing the hostile Action 3 while receiving a friendly Action 1 or 2 from the Alter agent, as is known from the Prisoner’s Dilemma.

Here, we examine a scenario in which Ego and Alter have their predefined preferences regarding the possible outcomes of their interaction. This set-up is characterized by two state variables *A* and *E*, each having three possible values, resulting in a total of nine environmental states. Figure 2 visualizes the environmental states and the agent’s preferences encoded in a prior distribution *P*(*S*|*m*) over the states. A hypothetical realization of a specific outcome is thus always connected with a surprise for the agent. In the example, the states E1 and E2 can be interpreted as a friendly commitment of Ego, whereas E3 can be interpreted as his commitment to being hostile. The matrix shows that Ego has a higher preference for a friendly commitment of Alter while being hostile himself. In this setup, the agents Ego and Alter can select policies to influence the states. The Ego agent has three policies that directly correspond to the environmental states, i.e., “Policy 1” results in *E=E1*, “Policy 2” results in*E* = *E*2 and “Policy 3” results in *E=E3*. For Alter it is analog.

The situation described here would correspond to the prisoner’s dilemma if the agents only had these three options and the preference matrix. In the prisoner’s dilemma, both agents have the tendency to select the hostile policy as a dominant strategy, resulting in an overall suboptimal outcome. One solution to the prisoner’s dilemma is the agreement on an enforceable contract (Fehr et al., 2002). In a utility maximization approach the agents could sign a contract, which makes the hostile policy (“Policy 3”) more expensive or even close to impossible (when the costs get extraordinarily high). There are also other solutions to this dilemma, for example if the game is repeated over time, as is the case with the “repeated prisoner’s dilemma.” Novak’s five rules, which relate to five mechanisms for the development of cooperation (selection of relatives, direct reciprocity, indirect reciprocity, reciprocity in the network, and group selection), each describe the needed payout structure and the probability of replay in terms of the repeated prisoner’s dilemma (Nowak, 2006). The statistical-physics perspective enables an interesting approach which has already been used as a basis for the free-energy principle and which can also be used for the game theoretical framework known as the repeated prisoner’s dilemma (Javarone, 2016, 2018).

**FIGURE 3:**
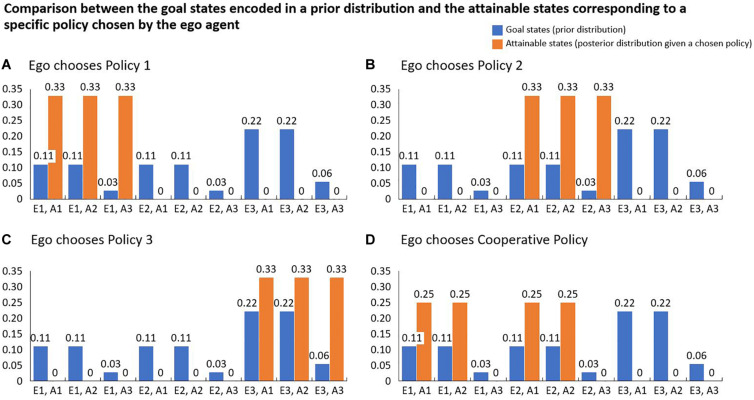
Every possible policy that the Ego agent can choose is characterized by a distribution of states that can be reached by using it. **(A–D)** contain the posterior distributions for the four possible policies Ego can choose from. Each of these distributions is then compared with the agent’s preference distribution using the Kullback-Leibler Divergence *D*_*KL*_. The agent selects the policy that has the minimal *D*_*KL*_ value.

In our example, however, the constellation is different in some respects from the classic and the repeated prisoner’s dilemma. We look at a one-time decision-making with enforceable contracts, where the agents minimize their expected surprise instead of maximizing a payout or utility. The agent’s choice to put an enforceable contract into place, makes the environmental states containing hostile commitments impossible. In combination with the policies described above the agents have now a vector of policies π={P′olicy 1,′`Policy 2,′P′olicy 3,′`Cooperate}′ to choose from in order to influence the environmental states *S*. The updated probability distribution over the states *S* given a specific policy represents the posterior probability distribution. In Figure 3, we visualize the posterior distributions for all of Ego’s potential policy choices. Figure 3A shows the posterior distribution given the policy choice *P*(*S*|π = *Policy* 1). In this example, Ego does not know about the preferences of the Alter agent and thus assumes equal probability for each of Alter’s actions.

**FIGURE 4:**
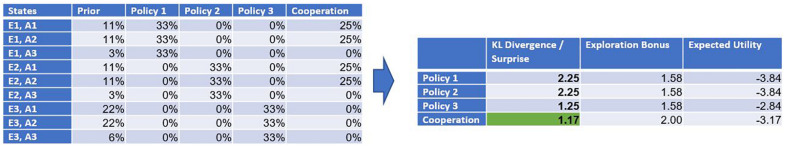
**(Left Table)** Summary of all probability distributions used in the situation. **(Right Table)** The resulting risk of each policy is calculated using the Kullback-Leibler Divergence *D*_*KL*_. The agent chooses the policy that has the smallest divergence, meaning that the distribution over the attainable states is closest to the agent’s preferences encoded in the prior distribution. In the situation described, the cooperative policy, which excludes the hostile actions for both agents, minimizes the risk for the Ego agent. Since the Alter agent is a copy of the Ego agent, it is likely that a cooperation is agreed upon.

We use this constructed example to illustrate, that cooperation (in the sense of a willingness to introduce and accept social rules) can also arise from a free energy perspective, according to which future surprises or risks are minimized. As stated above, calculating the risk (or future surprise) of a policy is done by calculating the Kullback-Leibler divergence between the prior distribution encoding the agent’s preferences and the posterior distribution over the attainable states given the chosen policy. Figure 4 contains the results of the calculations for all 4 possible policies. The table on the right side shows that minimizing future surprise would lead to a cooperation between the agents because the introduction of the social rule predicts the smallest future surprise.

In conclusion, we were able to show that the principle of minimizing surprise or free energy, which has proved successful in various fields including neuroscience, can also explain the emergence of social rules, when multiple agents communicate with each other. Of note, it can also explain why certain social rules or laws are not introduced, even if they would increase the expected utility.

### Limiting the Policy Repertoire May Also Increase Uncertainty

As mentioned above the risk of a policy can also be reformulated into the exploration bonus and an expected utility. The exploration bonus results in a higher preference for a policy that allows for multiple attainable states rather than focusing on only one relatively promising state.

In the example above only very hostile or cooperative actions existed. Policies excluding the very hostile actions therefore lead to surprise minimization. In setups with a more granular difference between the actions, it could be difficult to find the right criteria for a policy. A very restrictive policy could exclude so many options that the loss in exploration bonus is higher than the gain in the expected utility. In political policy design it can be observed that policies aim to leave room for individual decision making and reactions but try to avoid the most severe reactions. One example is the higher penalty for crimes of murder compared to misconducts of pedestrians crossing the street at a red light. If a policy maker would only consider maximum expected utility, he would punish all crimes very severely and uses prohibitions and bans in economics more often.

The surprise minimization approach values freedom of choice in the future and actively rewards policies, strategies or constraints that only exclude the most harmful states while preserving flexibility and self-determination.

### Stress Can Occur in Threatening Situations Where There Is no Clear Best Policy

**FIGURE 5:**
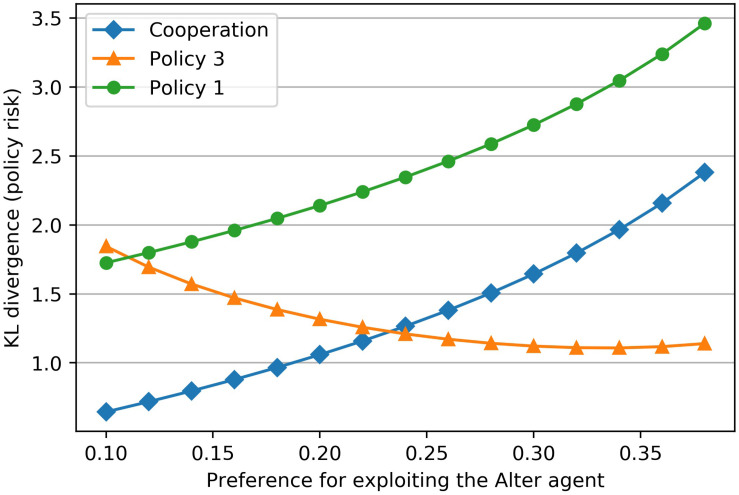
The preference for the Ego agent to exploit the Alter agent is encoded in Ego’s prior. Changing this preference for exploiting (*x*-axis) results in a change of the Kullback-Leibler Divergences of the different policies (*y*-axis). Up to a certain exploitation preference the agent will cooperate (dove-like behavior). Beyond that cut-off point a cooperation will not take place (hawk-like behavior). The cut-off point is marked by the intersection of the two policies “Cooperation” and “Policy 3.” At this point there is no clearly best policy because both policies have the same level of risk or future surprise.

The prior distribution, which encodes the goal states of the Ego agent in the above example, describes his preference to exploit the Alter agent. In this example, the preference is coded with a low to medium probability (*p* = 0.22) in the prior distribution. If we assign a higher (or lower) value to the reward for exploitation while keeping all other rewards constant, the probability for the states A3, A1 and A3, A2 increases (or decreases) in the prior distribution. Figure 5 shows that the Kullback-Leibler-Divergence for each policy depends on this exploitation reward. A very “bold” (hawk-like) agent with a high preference for exploitation (*p* = 0.35) will not cooperate in this example, while a relatively “cautious” (dove-like) agent (*p* = 0.15) will cooperate.

In evolutionary terms different organisms adopt different behavioral strategies. It has become clear that natural selection maintains a balance of different traits preserving genes for high aggression (hawk-like) and low aggression (dove-like) within a population. The existence of these personality types (Hawks–Doves) is widespread in the animal kingdom, not only between males and females but also within the same gender across species (Korte et al., 2005). Given the results of our analysis, the exploitative behavior of hawk-like agents and the cooperative behavior of dove-like agents appear to be due to a set of neurally-coded-prior probabilities that determine their different goal states.

The priors which determine individual goals are not necessarily fixed, but can also be influenced by the priors of other agents who live with him in the same society. Ethnographic records of 60 societies showed that the valence of several cooperative behaviors (including helping kin, helping your group, reciprocating, and dividing disputed resources) is uniformly positive (Curry et al., 2019). Such a social consensus can make the friendly policies more attractive for the Ego agent, reduce his preference for exploiting the Alter agent, lower the cut-off for not cooperating, and thus make the Ego agent more likely to cooperate. If one lives in a society that values cooperation highly, cooperation is indeed not a bad decision to avoid surprises.

In our example, there is also the case of a prior distribution with a medium preference for exploitation (*p* = 0.24), where the Kullback-Leibler divergence for cooperation and non-cooperation is equal. In this case, the medium preference for exploitation of the counterpart equals the cut-off point for abstaining from cooperation. In such a situation the agent has no clear best policy available. When this choice of policy is uncertain in a situation that endangers well-being, stress arises.

Such a stress reaction does not only occur in critical situations where the decision to cooperate or not to cooperate has to be made. Generally speaking, people who feel threatened by changes in their external environment or in their internal body environment may find themselves confronted with this question: *“What policy should I select to safeguard my future physical, mental and social wellbeing?”* According to a novel definition, “stress” arises in those people who are uncertain about the answer (Peters and McEwen, 2015). In other words, stress occurs when expectations are anticipated to be disappointed when using any of the available policies. The uncertainty that arises when it turns out that all available policies are equally risky initiates a physiological uncertainty resolution program (Peters et al., 2017).

As the ACC plays a key role in reducing uncertainty, it may disinhibit (if necessary) the amygdala, which is the center of the stress response network. The amygdala in turn activates three components of the stress response. First, the locus coeruleus in the brain stem initiates the release of noradrenaline from nerve endings that reach almost every part of the brain, particularly in the cerebral cortex (Aston-Jones and Cohen, 2005). Here, norepinephrine accelerates the information transmission from one neuron to another, thereby increasing the bit rate per second and also the energy consumption of the brain. Secondly, activation of the sympathetic nervous system (SNS) leads to extra glucose being supplied to the brain (Peters et al., 2004). This supplementary glucose is actively withdrawn from the body stores (Peters and Langemann, 2009). The extra glucose can cover the brain glucose requirement for enhanced information processing. Third, the activation of the hypothalamic-pituitary adrenal axis (HPA) leads to the release of the stress hormone cortisol from the adrenal glands. Cortisol acts throughout the human organism, including the brain, where it modulates how successful policies are learned (McEwen and Morrison, 2013; Peters et al., 2018). Overall, the stress response with its three components aims to eliminate the uncertainty as quickly as possible (Peters et al., 2017). It is obvious that the uncertainty resolution program is adaptive and has clear advantages, although people feel uncomfortable while it is operating.

At this point we come back to Ego’s decision whether to cooperate or not. If the two agents do not yet know each other, the Ego agent may assume that all reactions of the Alter agent are equally likely, which is not necessarily true. If the Ego agent is uncertain about how to decide, he could try to better predict the Alter agent’s goals with the additional resources available to him during stress.

## Discussing Potentials of Surprise Minimization for Sociology and Medicine

We have shown for two agents that free energy or surprise minimization can explain the emergence of social rules. In the famous Prisoner’s Dilemma, maximization of expected utility can also explain the emergence of rules, so why should we use the free-energy principle to explain a similar outcome? We have four good reasons.

1. Experimental evidence shows that surprise minimization can explain human decision making better than utility maximization.
2. The freedom of choice has an explicit term in the surprise minimization.
3. The free energy principle can explain not only previously unexplained phenomena such as the so-called “binocular rivalry,” but – as a more general framework – even cooperation between two agents.
4. Using the free energy principle, research has shown how uncertainty in decision-making can lead to toxic stress with secondary diseases such as coronary heart disease, depression and type 2 diabetes. Restrictive policy designs can easily put an individual in an uncertain decision-making situation where all his or her policies seem unsuitable due to the lack of open options. In contrast to utility maximization, surprise minimization can explain why restrictive policy designs can put large parts of society under toxic stress.

### Ad 1: Surprise Minimization Explains Experimental Data Better Than Utility Maximization

Minimizing surprise makes a prediction at variance with expected utility models; namely, that in addition to attaining valuable states, agents attempt to “keep their options open.” Schwartenbeck et al. (2015) tested this prediction using a simple binary choice paradigm and showed that human decision-making is better explained by surprise minimization compared to utility maximization. These results illustrate a limitation of purely economic motivations in explaining choice behavior and instead emphasize the additional importance of belief-based motivations aimed at keeping as many options open as possible.

### Ad 2: Freedom of Choice Explicitly Formulated

As mentioned above, the free energy principle optimizes the sum of exploration bonus (i.e., the entropy over attainable states) and expected utility. Thus, surprise minimization is not opposed to the classical maximization of expected utility, but surprise minimization is actually an extension of utility maximization.

### Ad 3: Wide Range of Validity of the Surprise Minimization – Binocular Rivalry

A growing body of experimental evidence supports the free-energy principle as it is formulated in concept of the Bayesian brain (Hohwy, 2013; Clark, 2016). The Bayesian view conceives perception as a process of top-down predictions, which are corrected via bottom-up prediction errors. In contrast, the conventional view of perception says that the information flow is only directed bottom-up, e.g., from the retina via the thalamus to the visual cortex.

A classic experiment challenges the conventional view (Hohwy et al., 2008): Test persons are put on virtual reality glasses (i.e., glasses with small monitors instead of lenses). One eye is shown the image of a house, at the same time the other eye is shown the image of a face. What is striking is that the test person initially perceives only one image, e.g., the house for about eight seconds; and then only the face for eight seconds; and then the house again, and so on. The two monocular images compete with each other, causing repetitive perceptual changes. This counter-intuitive effect is referred to as binocular rivalry.

The conventional view has difficulties to explain these experimental results. For, during the rivalry, the physical stimulus in the world remains the same and yet the perception alternates. However, the Bayesian brain perspective can provide an explanation: There are three relevant candidate hypotheses to explain what may have caused this unusual sensory input: it is a house only, it is a face only, or it is a face-house blend. The system will select one of these hypotheses based on their likelihood (how likely it is that a house, face, or face-house blend would have caused this input) and secondly their prior probability (how likely it is that one should be seeing a house, a face, or a face-house now, irrespective of the actual sensory input). In the hierarchical brain structure, the priors on the lower levels of abstraction encode details (e.g., nose, eyes, and roof tiles), the priors on the higher levels encode rules and principles (e.g., it is unlikely that two things coexist in the same place at the same time). The combined face-house-blend hypothesis has the highest likelihood. This is so, because it accounts for more of the sensory input than the face or the house hypothesis on their own. Thus, at a certain level in the Bayesian hierarchy, the face-house-blend hypothesis would have the highest prior probability. On the level above, however, the coexistence of face and house has an extremely low prior probability. This extremely low prior probability at the higher level of abstraction overrides the prior belief at the level below. In this way, it is either the face or the house hypothesis that is selected and determines alternating perception (Hohwy et al., 2008). There were many other neuroscientific experiments that gave an enormous boost to the Bayesian approach (Hohwy, 2013; Clark, 2016). Today, inference to the best explanation clearly supports the free-energy based Bayesian brain concept.

Through the method of surprise minimization, human decision-making can be anchored in a much more fundamental biological principle, the free-energy principle, which can explain many phenomena in the life sciences, especially those observed in brain research, such as action, perception and learning (Friston, 2010; Ramstead et al., 2018). As shown in this paper, even the decision whether to agree on a social rule with someone else can be derived from surprise minimization.

### Ad 4: Policy Makers Can Reduce Toxic Stress in Society by Weighing up the Expected Utility and Open Options for the Individual

As mentioned above, the physiological stress response in agents can be seen as an uncertainty-reduction program that puts the brain into a highly active (hypervigilant) mode that helps to gather more information as quickly as possible to finally determine one best policy (Peters et al., 2017). The agent experiences an episode of “good stress” if he/she is able to resolve the uncertainty.

Toxic stress, however, occurs when it is not possible to resolve the uncertainty and this situation persists for years, so that the agent becomes frustrated by the futile search for a successful policy (Peters and McEwen, 2015). In such a case, the brain gets into a hypervigilant state day and night (Hermans et al., 2011). Therefore, the brain’s average energy demand is increased in the long run. Such an energetic overload of brain metabolism has long-term consequences: First, chronic hyperactivity of the amygdala leads to typical depression (McEwen, 2012). Secondly, the chronically high energy requirement of the brain leads to intermittent or permanent SNS activation and thus to an increase in the average heart rate (Jones et al., 2011). An increased cardiac output upturns the cerebral energy supply, but also the probability of turbulences occurring at bifurcations and branching points in the arterial system (Malek et al., 1999). If turbulences appear chronically, atherosclerosis develops at these predilection sites (Chatzizisis et al., 2007). In the long run, toxic stress causes myocardial infarction and stroke (Orth-Gomer et al., 2009; Gulliksson et al., 2011).

If the agent is stuck in such a difficult situation and cannot resolve uncertainty himself, external help in the form of psychological or psychiatric intervention may become necessary. But there are also other cases where individual support is not sufficient to reduce uncertainty. Social factors such as social inequality (e.g., low level of education) generate uncertainty and stress, which in turn promote the development of the above-mentioned diseases of civilization. Experimental social interventions have been shown to improve uncertainty, type 2 diabetes mellitus and obesity (Ludwig et al., 2011; Ludwig et al., 2012). Therefore, socio-political policies are also necessary to reduce people’s uncertainty.

In summary, uncertainty in the choice of policy arises when, in a threatening situation, all available individual policies appear dangerous (little expected utility) and leave little room for freedom of choice (exploration bonus). In view of the dramatic consequences of uncertainty and toxic stress, policy makers should carefully weigh up expected utility and the individual’s freedom of choice when designing legislation.

## Conclusion

This paper extends the role of the surprise minimization principle to social contexts where multiple agents are involved. We could show that cooperative behavior and social rules can emerge from surprise minimization. Our analysis has shown that an agent who has to decide whether to cooperate or not can assess the risks of both options. The risk of each option depends largely on how pronounced the agent’s preference is for exploiting the other agent. There is a cut-off point below which the preference to exploit is low enough that cooperation is expected to produce minimal surprises, and above which the preference is high enough that non-cooperation is more favorable in this respect. Of course, it has been shown that classical utility maximization can explain cooperative behavior too. However, surprise minimization seems to be superior to the classical method of utility maximization for four reasons: First, surprise minimization, unlike utility maximization, predicts that, in addition to achieving high utility states, agents will try to “keep their options open.” This prediction was tested using a simple binary choice paradigm, and it became evident that human decision making can best be explained by surprise minimization. Secondly, freedom of choice (keeping options open) is explicitly formulated as “the entropy of future states.” Third, surprise minimization is a more general principle that can best predict observations in many areas of biology, including the neurosciences (e.g., binocular rivalry). Fourth, surprise minimization – but not classical utility maximization – can be used to explain why policy makers who carefully balance utility maximization and freedom of choice can reduce toxic stress in human societies. In all, our analysis reveals that the surprise minimization principle can be regarded as a fundamental framework that can also be applied to the emergence of cooperation and social rules.

One direction for further research would be to investigate a more complex setting in which agents interact, involving several time steps, e.g., with the possibility of withdrawing from social rules or changing preferences. Another direction for further development is to examine whether “keeping options open” is also important for the other forms of cooperation with more than two actors described by the Nowak rules, such as indirect reciprocity, network reciprocity or group selection.

## Author Contributions

AP had the conceptual idea and wrote the first draft of the manuscript. MH worked on the manuscript, set-up the example calculations, and the visualizations. Both authors contributed to manuscript revision and approved the submitted version.

## Conflict of Interest

The authors declare that the research was conducted in the absence of any commercial or financial relationships that could be construed as a potential conflict of interest.
